# Association between Neighborhood Environment and Quality of Sleep in Older Adult Residents Living in Japan: The JAGES 2010 Cross-Sectional Study

**DOI:** 10.3390/ijerph17041398

**Published:** 2020-02-21

**Authors:** Michiko Watanabe, Yugo Shobugawa, Atsushi Tashiro, Asami Ota, Tsubasa Suzuki, Tomoko Tsubokawa, Katsunori Kondo, Reiko Saito

**Affiliations:** 1 Division of International Health, Graduate School of Medical and Dental Sciences, Niigata University, 1-757 Asahimachi dori, Chuo-ku, Niigata city, Niigata 951-8510, Japan; miwatanabe@n-seiryo.ac.jp (M.W.); tsubokawa@n-seiryo.ac.jp (T.T.); jasmine@med.niigata-u.ac.jp (R.S.); 2 Department of Nursing, Faculty of Nursing, Niigata Seiryo University, 1-5939, Suidocho, Chuo-ku Niigata 951-8121, Japan; 3 Department of Active Aging (donated by Tokamachi city, Niigata Japan), Graduate School of Medical and Dental Sciences, Niigata University 1-757 Asahimachi dori, Chuo-ku, Niigata city, Niigata 951-8510, Japan; 4 Niigata City Public Health Center, 3-1-1, Shichikuyama, Chuo-ku Niigata 950-0914, Japan; a.tashiro11@city.niigata.lg.jp; 5 Division of Health and Nutrition, University of Niigata Prefecture, 471 Ebigase Higashi-ku, Niigata city, Niigata 950-8680, Japan; asammy@unii.ac.jp; 6 Niigata Health Service Center, 2-180-5 Hakusanu-ura, chuo-ku, Niigata-shi, Niigata 951-8680, Japan; tsubasa.szk@gmail.com; 7 Center for Preventive Medical Sciences, Chiba University, 1-8-1 Inohana, Chuo-ku, Chiba city, Chiba 260-8670, Japan; kkondo@chiba-u.jp; 8 Department of Gerontology and Evaluation Study, Center for Gerontology and Social Science, National Center for Geriatrics and Gerontology, 7-430 Morioka-cho, Obu City, Aichi 474-8511, Japan

**Keywords:** multilevel Poisson regression, older adults, physical environment, sleep quality, social environment

## Abstract

Poor sleep is associated with lifestyle, however, few studies have addressed the association between sleep quality and the neighborhood environment. This study aimed to investigate the associations between living environment factors and sleep quality in older people. Participants were community-dwelling people aged ≥65 years who participated in the 2010 Japanese Gerontological Evaluation Study. The data of 16,650 people (8102 men, 8548 women) were analyzed. Sleep quality (good or poor) was evaluated using a self-administered questionnaire. Multilevel Poisson regression analysis stratified by depressive status (measured by the Geriatric Depression Scale-15 [GDS]) was conducted with sleep quality as the dependent variable and social and physical environmental factors as explanatory variables. The 12,469 non-depressive respondents and 4181 depressive respondents were evaluated. The regression analysis indicated that non-depressive participants slept better if they lived in environments with few hills or steps (prevalence ratio [PR] = 0.75, 95% CI: 0.56–0.9) and with places where they felt free to drop in (PR = 0.51, 95% CI: 0.26–0.98). For depressive participants, these associations were not evident. Living alone, poor self-rated health, low income, and unemployment were associated with poor sleep quality. In addition to support with these individual factors, improving environmental factors at the neighborhood level may improve the sleep quality of community-dwelling older adults.

## 1. Introduction

Sleep is one of the most important lifestyle factors for maintaining a good health status, alongside diet and exercise. Poor sleep causes lifestyle-related diseases such as hypertension, diabetes mellitus, or metabolic syndrome [1,2,3] and even psychiatric diseases such as depression [4,5,6]. In addition, it has been recently elucidated that poor sleep is associated with cognitive impairment and the onset of dementia in older adults [7,8,9].

One in five Japanese persons experiences sleep problems, and this proportion is increased to one in three persons in the older population. Poor sleep is a serious problem in older adults. The duration of sleep is generally longer in old age than in young age, but the quality of sleep is reduced due to light sleep, interrupted sleep, and/or early awakening, which then cause sleepiness during the day and declining activity [10]. Additional causes of poor sleep such as insomnia are particularly prevalent in older adults. For instance, negative life events such as retirement, bereavement, and living alone may be psychiatric stressors [11], which cause sleep disorders. Consequently, such persons lose social roles and their physical and mental activity is reduced [12]. Finally, they tend to be affected by physical and mental diseases [13]. Such negative cycles can occur in older adults.

Social determinants of health have become topics of study in recent years, and it has become clear that some factors that are not amenable to improvement by individual efforts alone also have an effect on health. The World Health Organization (WHO) Adelaide Statement on Health in All Policies issued in 2010 emphasized that government objectives are best achieved when all sectors include health and well-being as key components of policy development [14]. In light of this reality, there is an increasing need to create local environments that encourage people to remain healthy as they age. In 2011, the WHO set up the WHO Global Network of Age-friendly Cities and Communities with a focus on social determinants of health. Baglioni et al. [4] and Pugh et al. [15] have suggested that the neighborhood environment may affect the functional health of older people. There is thus an increasing awareness that health support must involve not only support at the individual level, but also community-level support.

Most previous studies of sleep have addressed its association with individual factors such as sex, age, income, and educational achievement [16,17]. In terms of the association between sleep and community-level environmental factors that affect individuals, a few studies have investigated the associations of public order and social capital with sleep at the individual level [18,19,20]. However, no study has yet addressed the association between sleep quality and the neighborhood physical environment using multilevel analysis.

Because older people spend the vast majority of their time in residential neighborhoods, they are highly susceptible to the impact of the local environment in the neighborhood. In addition to public order and social capital, the physical environment may also affect sleep in older people. Therefore, in this study, we aimed to perform a multilevel analysis of the association between environmental factors in Japanese residential neighborhoods and sleep quality.

## 2. Materials and Methods

### 2.1. Participants

The subjects of this study were participants of the Japanese Gerontological Evaluation Study (JAGES) in 2010 [21]. Participants were community-dwelling people aged ≥65 years who lived in 31 municipalities in 11 of the 47 prefectures in Japan. Social and physical environments surrounding the participants varied by municipalities: for instance, socially between tight bonding communities and those with less bonding; and physically between highly populated urban cities and less populated rural areas. Such environmental variety affects lifestyle and even physical and mental health outcomes which can cause health disparities [22]. The participants were not certified to need long-term care. A self-administered questionnaire was distributed by postal mail to each of the 160,382 eligible participants between August 2010 and January 2012. The participants primarily responded to the questions by themselves. The questionnaire included five modules, and each module was sent randomly to one-fifth of the participants. Items to evaluate sleep quality were included in one of the five modules.

### 2.2. Definition of Sleep Quality

Sleep quality (good or poor) was evaluated on the basis of the participant’s response to the self-administered questionnaire. The quality domain from the Pittsburgh Sleep Quality Index (PSQI) was applied to evaluate sleep quality. The PSQI sleep quality item asks “During the past month, how would you rate your sleep quality overall?” with possible responses: “very good”, “fairly good”, “fairly bad”, and “very bad”. This single item was selected as the total PSQI score and incorporates information of other domains of sleep in the PSQI [23] (e.g., sleep timing and continuity). In addition, the four responses were dichotomized into two values, good (very good or fairly good) or poor (fairly bad or very bad) and were used as the dependent variable.

### 2.3. Explanatory Variables

#### 2.3.1. Individual-Level Variables

We evaluated the association between sleep quality and the following individual-level factors: sex; age (65–69, 70–74, 75–79, 80–84, ≥85 years); living alone (yes, no); self-rated health (good, poor); employment (yes, no); equalized household income (<2 million yen, 2–3.99 million yen, ≥4 million yen); education (<6 years, 6–9 years, 10–12 years, ≥13 years); daily walking time (<60 min, ≥60 min); and any medical treatments received for conditions other than sleep disorders such as hypertension, diabetes, respiratory diseases, cancer, heart disease, or any diseases. Depressive status (non-depressive or depressive) defined by the Geriatric depression scale (GDS)-15 (GDS score of <5: non-depressive or ≥5: depressive) was also included as an individual variable in the main analysis. The respondents were stratified into two groups: individuals with a depressive trend as defined by a GDS score of ≥5, and those without a depressive trend as defined by a GDS score of < 5 [24,25].

#### 2.3.2. Neighborhood-Level Variables

According to a previous study, neighborhood environment was evaluated by two components, the social environment and physical environment [26]. For evaluation of the social environment, we applied a modified version of health-related social capital indices [27], which are composed of the following three dimensions: (1) civic participation, (2) social cohesion, and (3) reciprocity. Civic participation was scored by asking participants whether or not they took part in a volunteer group, sports group, or hobby activity at least once per month or less than once per month, and calculating the rate of participation in each group in the school district. Civic participation was therefore scored as follows: (rate of volunteer group participation × 0.6) + (rate of sports group participation × 0.8) + (rate of hobby activity × 0.9). Social cohesion was scored by asking respondents about community trust, norms of reciprocity, and community attachment on a five-point scale (“strongly agree”, “somewhat agree”, “neither agree nor disagree”, “somewhat disagree”, and “completely disagree”), with the responses “strongly agree” and “somewhat agree” categorized as “agree” and “neither agree nor disagree,” “somewhat disagree,” and “completely disagree” categorized as “disagree,” and calculating the rate of “agree.” Social cohesion was therefore scored as follows: (rate of “agree” to community trust × 0.9) + (rate of “agree” to norms of reciprocity × 0.8) + (rate of “agree” to community attachment × 0.7). Reciprocity was evaluated by asking whether or not participants received emotional support, to whom they were providing emotional support, and from whom they were receiving instrumental support, with the responses categorized as yes or no, and calculated the rate of “yes.” Reciprocity was therefore scored as follows: (rate of “yes” to receiving emotional support × 0.8) + (rate of “yes” to providing emotional support × 0.8) + (rate of “yes” to receiving instrumental support × 0.7).

Regarding the surrounding physical environment, eight items were evaluated. We asked whether the respondents had each of following environmental items within 1 km of their residence: (1) “Locations with noticeable graffiti or undisposed garbage”; (2) “Parks or foot paths suitable for exercise or walking”; (3) “Locations difficult for walking, such as hills or steps”; (4) “Roads or crossroads with a great risk of traffic accidents”; (5) “Fascinating views or buildings”; (6) “Shops or facilities selling fresh fruits and vegetables”; (7) “Dangerous places when walking alone at night”; and (8) “Houses or facilities where you feel free to drop in.” The potential responses were “Many”, “Some”, “Few”, “None”, and “I don’t know.” The five responses were dichotomized into two values: Yes (“Many” or “Some”) or No (“Few” or “None” or “I don’t know”), and used as explanatory variables.

### 2.4. Statistical Analysis

Many previous studies have reported a significant association between sleep and depression [18,20,26]. Thus, we stratified the responses by depressive status to evaluate sleep quality after testing the potential interactions between depressive status and each of the explanatory variables on sleep quality.

First, the baseline characteristics of the respondents were stratified by depressive status (GDS score of <5 or ≥5). Univariate analyses using the chi-square test were used to evaluate the associations between sleep quality and each of the individual characteristics. Data missing the outcome variable or GDS score were excluded from the analysis. If data were missing other explanatory variables, the corresponding observation was assigned to the category of the missing variable [28]. The threshold for statistical significance was set at P-value (*p*) < 0.05 in a two-tail test.

Second, neighborhood characteristics in the school districts were evaluated. The school district was used as the neighborhood unit, which is a proxy for a geographical area that is easy for older adults to navigate [29]. Social capital indices in each school district were calculated. Applicable rates for the eight items of the surrounding physical environment were also calculated at each school district unit. Third, a multivariate analysis was conducted to explore the factors associated with sleep quality status including neighborhood-level factors using a multilevel Poisson regression model. To avoid overestimation of odds ratios with logistic regression analysis [30], since the proportion of people with poor sleep was >10%, we used a Poisson regression model with strong dispersion. We conceptualized the analysis in a multilevel structure, comprising individual factors (individual-level) and nested within school district factors (neighborhood-level). We fitted the data using multilevel Poisson regression procedures with a random intercept model, adjusting for both individual and neighborhood levels as fixed effects and setting sleep quality as the dependent variable. The method of estimation was a restricted maximum likelihood procedure. The first set of analyses involved estimating the null model (Model 1). The null model allows for the decomposition of variance in sleep quality to determine whether it was attributable to neighborhood-level and between-person variation. Next, the modeling was performed in three steps: Model 2, only individual-level factors were added; Model 3 had both individual-level factors and neighborhood-level social environmental factors; and Model 4 had both individual-level factors and neighborhood-level surrounding physical environmental factors. The fixed effect results are presented as prevalence ratios (PRs) with 95% confidence intervals (CIs). The random effect results are presented as neighborhood-level random variance with standard error (SE). The calculated proportional changes in variance (PCV). Statistical analyses were performed using STATA version 14 (StataCorp, College Station, TX, USA).

### 2.5. Ethical Considerations

The study protocol for the JAGES project was approved by the Ethics Committee of Nihon Fukushi University (No. 10-05). A letter informing all potential participants of the ethical considerations, including the study methods, was enclosed with the survey, and the return of the completed survey questionnaire was considered to indicate the provision of informed consent.

## 3. Results

### 3.1. Characteristics of the Respondents

**Figure 1:**
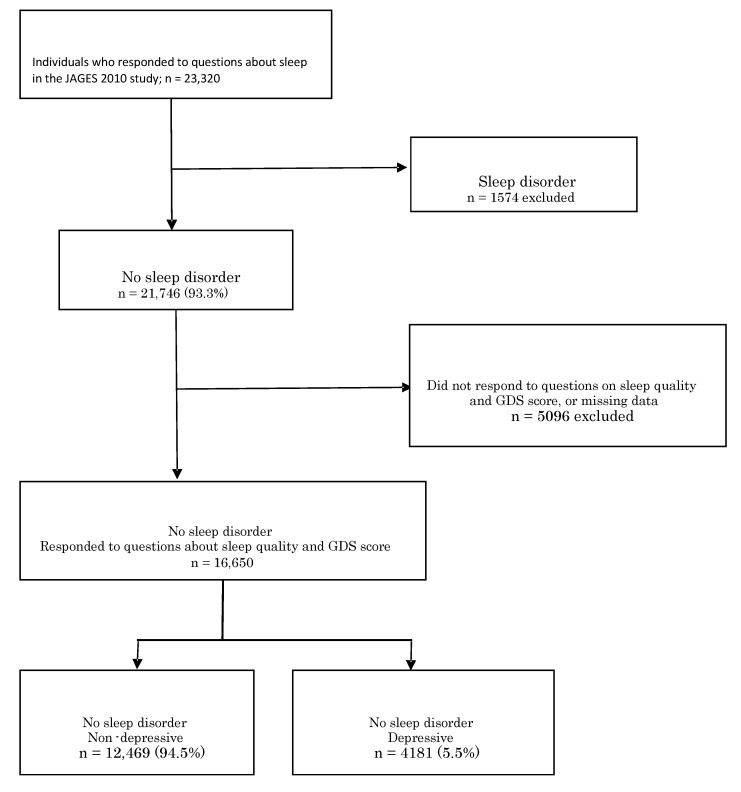
Flow chart of the enrollment process of the study participants. People with sleep disorders (n = 1574) and those who did not respond to the sleep quality module (n = 5096) were excluded. Finally, the data of 16,650 people (12,469 without depressive status, 4181 with depressive status) were analyzed.

A total of 106,460 people responded to the survey (response rate, 66.4%; Figure 1). Among the respondents, 23,320 were sent the module (module “D”) that included items related to sleep quality to be analyzed. Among the eligible respondents, people with sleep disorder (n = 1574) and those who did not respond to the sleep quality and GDS score module (n = 5096) were excluded. A question which asks about current diseases in the questionnaire could identify sleep disorders (e.g., insomnia or snoring). Finally, the data of 16,650 people (8102 men, 8548 women) were analyzed.

**Figure 2:**
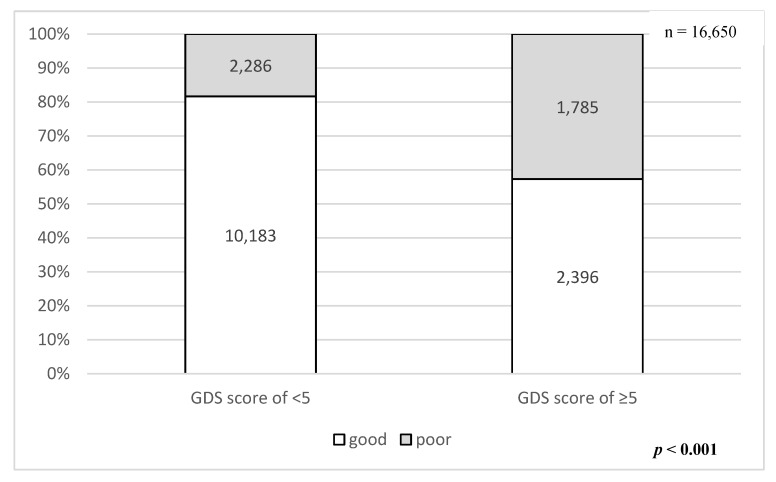
Prevalence of poor sleep between depressive and non-depressive participants. The rate of poor sleep in depressive participants (Geriatric Depression Scale [GDS] score of ≥5) was significantly higher than that in non-depressive participants (GDS score of <5) (*p* < 0.001).

The average age of all respondents was 73.9 ± 6.2 years (range 65–101 years). The baseline characteristics of the respondents are provided in Table 1. Among the 12,469 non-depressive respondents (GDS score of <5), 2286 (18.3%) had poor sleep. Of the 4181 depressive respondents (GDS score of ≥5), 1785 (43.3%) had poor sleep. The rates of poor sleep significantly differed between non-depressive and depressive respondents (*p* < 0.001; Figure 2).

In the non-depressive respondents, the proportion of women with poor sleep was significantly higher (55.8%) than the proportion of women with good sleep (50.6%; *p* < 0.001). Poor sleepers tended to be younger than good sleepers (*p* < 0.001). The rates of living alone, poor self-rated health, and not working were respectively higher in poor sleepers than in good sleepers (all *p* < 0.001). Regarding socioeconomic status, lower equivalent income and shorter educational attainment were observed more frequently in poor sleepers (both *p* < 0.001). Walking time was significantly shorter in poor sleepers than in good sleepers (*p* < 0.001). In addition, the rate of people who has any co-morbidity was higher in poor sleepers than in good sleepers (*p* < 0.001).

At the individual level, there was no association between poor sleep quality and civic participation in volunteer, sports, or hobby groups. In contrast, low scores for community trust, norms of reciprocity, and community attachment were significantly associated with poor sleep quality (*p* < 0.001). This result was the same for both depressive and non-depressive respondents. Non-depressive respondents who were not receiving instrumental support had significantly poorer sleep quality (*p* < 0.007). In depressive respondents, those who were not receiving emotional support nor receiving instrumental support had significantly poorer sleep quality (Table 2).

### 3.2. Variety of Sleep Quality among the Neighborhood Level

The percentage of poor sleepers ranged from 9.0% to 47.0% among the 568 school districts. There was significant variation in sleep quality between communities: community-level variance was 0.00045 in whole respondents (Model 1 in Table 3) and 0.00074 in non-depressive respondents (Model 1 in Table 4). However, there was no significant variation between communities for depressive respondents; community-level variance was $4.6\times 10^{-21}$ (Model 1 in Table 5). The calculated proportional changes in variance (PCV) are shown in each table, which indicate community-level variance due to neighborhood social capital or objective built environment. PCV values are not shown in Table 5 because community-level variance was almost zero for depressive respondents.

### 3.3. Individual and Neighborhood Factors Associated with Sleep Quality

For some of the neighborhood-level variables, there were significant interactions with depressive status on sleep quality.

#### 3.3.1. Whole Respondents

Model 2 revealed that higher GDS score of ≥5 (depressive) had poor sleep quality (PR of poor sleep: 1.93, 95% CI: 1.80–2.07) and older participants tended to have better sleep quality than younger participants (PR of poor sleep in those aged 75–79 years: 0.82, 95% CI: 0.75–0.90; 80–84 years: 0.74, 95% CI: 0.66–0.83; and ≥85 years: 0.68, 95% CI: 0.58–0.80; each compared to those aged 65–69 years) (Table 3). In addition, being female, living alone, poor self-rated health, and not working were significantly associated with poor sleep (each PR was 1.12, 95% CI: 1.05–1.19 [against being male]; 1.15, 95% CI: 1.05–1.26 [against not living alone]; 1.67, 95% CI: 1.55–1.80 [against good self-rated health]; and 1.11, 95% CI: 1.02–1.21 [against working]). A higher equivalent household income was significantly associated with good sleep (PR for ≥4.00 million yen: 0.83, 95% CI: 0.74–0.94 [compared to <2.00 million]); however, education attainment was not associated with sleep quality. Longer walking time (>60 min) was associated with good sleep (PR: 0.91, 95% CI: 0.85–0.98 [compared to <60 min]).

Model 3 revealed no significant associations between neighborhood-level social capital in any of the three components (civic participation, social cohesion, or reciprocity) and sleep quality. However, regarding the physical environment, fewer difficult locations for walking such as steps or slopes was marginally associated with fewer poor sleepers (PR = 0.84, 95% CI: 0.68–1.04) according to model 4. Similarly, more places (houses or facilities) where participants feel free to drop in were associated with fewer poor sleepers (PR = 0.59, 95% CI: 0.80–0.94).

#### 3.3.2. Non-Depressive Respondents (GDS Score of <5)

Model 2 revealed that older participants tended to have better sleep quality than younger participants (PR of poor sleep in those aged 75–79 years: 0.75, 95% CI:0.66–0.85; 80–84 years: 0.71, 95% CI: 0.6–0.83; and ≥85 years: 0.52, 95% CI: 0.4–0.68; each compared to those aged 65–69 years) (Table 4). In addition, being female, living alone, poor self-rated health, and not working were significantly associated with poor sleep (each PR was 1.16, 95% CI: 1.06–1.27 [against being male]; 1.16, 95% CI: 1.01–1.33 [against not living alone]; 1.97, 95% CI: 1.76–2.20 [against good self-rated health]; and 1.16, 95% CI: 1.04–1.29 [against working]). A higher equivalent household income was significantly associated with good sleep (PR for ≥4.00 million yen: 0.82, 95% CI: 0.71–0.95 [compared to <2.00 million]); however, education attainment was not associated with sleep quality. Longer walking time (>60 min) was associated with good sleep (PR: 0.88, 95% CI: 0.8–0.96 [compared to <60 min]).

Model 3 revealed no significant associations between neighborhood-level social capital in any of the three components (civic participation, social cohesion, or reciprocity) and sleep quality. However, regarding the physical environment, fewer difficult locations for walking such as steps or slopes was associated with fewer poor sleepers (PR = 0.75, 95% CI: 0.56–0.99) according to model 4. Similarly, more places (houses or facilities) where participants feel free to drop in were associated with fewer poor sleepers (PR = 0.51, 95% CI: 0.26–0.98).

#### 3.3.3. Depressive Respondents (GDS Score of ≥5)

In the depressive respondents, older participants tended to have better sleep quality than younger participants (PR of poor sleep in those aged 80–84 years: 0.79, 95% CI: 0.67–0.94; and ≥85 years: 0.83, 95% CI: 0.68–1.01; each compared to those aged 65–69 years) (Table 5). Sex differences were not observed with regard to sleep quality. Living alone was marginally associated and poor self-rated health was significantly associated with poor sleep (each PR was 1.14, 95% CI: 1–1.29 [against not living alone]; 1.49, 95% CI: 1.35–1.65 [against good self-rated health]). Neither equivalent household income nor education attainment was associated with sleep quality. Walking time and the existence of any diseases were not associated with sleep quality.

Model 3 revealed no significant associations between neighborhood-level social capital in any of the three components (civic participation, social cohesion, or reciprocity) and sleep quality, as was the case in the non-depressive respondents; Model 4 revealed there was no association between any physical environment factors and sleep quality.

## 4. Discussion

In this study, we investigated the associations between sleep quality and factors at the individual and neighborhood levels, using data from the JAGES 2010 study of older people in 31 Japanese municipalities. The analysis utilized large-scale data from over 100,000 survey respondents. We found that older people slept better if they lived in environments where there were places (houses or facilities) that they felt free to drop in. Because sleep quality is closely associated with depression, [4,5,6] we stratified the analysis by depressive status after analyzing the whole dataset. We found that non-depressive older people slept better if they lived in environments with few hills or steps and where there were places (houses or facilities) that they felt free to drop in. For depressive older people, these associations were not evident. The associations between individual-level factors and sleep quality were very similar to those described in previous studies, with living alone, poor self-rated health, low income, and unemployment being associated with poor sleep quality in older people [31,32].

The existence of places (houses or facilities) where older people feel free to drop in is believed to encourage them to go outside [33]. Going out not only increases their activity level but enables older people to engage in communication, such as enjoying conversations at these facilities when they drop in. It is possible that a satisfying lifestyle during the day may affect sleep quality.

It has previously been reported that physical activity increases significantly in a good neighborhood environment [34]. A residential environment with few hills or steps may make it easier for older people to go for walks [35]. A walkable environment is more likely to encourage older people to go outside, for example, to shop for daily necessities or to go for a walk or take other forms of exercise [36]. Making it easier to go outside may increase physical exercise, resulting in an appropriate level of fatigue and leading to good-quality sleep [21,37,38]. Although the existence of parks or foot paths might also promote physical activity, there is no significant effect in this dataset. One possible reason for this lack of effect is that people need to intentionally go to a park or foot path for exercising. In contrast, a residential environment with few hills or steps directly and unconsciously affects their habit of daily exercise. Such circumstances that promote physical activity without clear intention may sometimes successfully achieve the goal.

However, no such significant association with environmental factors was evident in depressive respondents in the stratified analysis. The absence of an association with physical environmental factors in depressive individuals may be because these individuals tend to isolate themselves and do not go out [39], making them less likely to be affected by the physical environment of the neighborhood. This might be a reasonable result.

According to the 2019 White Paper on the Aging Society (published by the Cabinet Office) [40], the Japanese population is currently aging rapidly, with 27.7% aged ≥65 years, the highest in any developed country. By 2065, this proportion is projected to reach 38.4%, with approximately one in every 2.6 people aged ≥65 years. According to the 2016 National Health and Nutrition Survey in Japan, 48.4% of all households included an older person aged ≥65 years, of which >50% were households consisting only of older people [41]. Even in households that include younger members, older people are often left by themselves during the day, and an environment in which they can easily go outside and that contains venues where they can communicate with others during the day will be one that is reassuring for them. Being able to spend time in a reassuring environment during the day may result in good quality sleep. Non-depressive individuals accounted for 75% of the respondents in this study. We believe that it is necessary to create an environment in which they can sleep better based on our study results.

Sleep problems are closely associated with individual-level factors such as individual lifestyle habits, social status, and income [16,17]. The results of our study suggested that in addition to support with these individual factors, approaching environmental factors from further upstream at the neighborhood level from the perspective of social determinants of health may also help to improve the sleep quality of local residents.

Because we considered that the local environment includes the human environment as well as the physical environment, we conducted an analysis of the former in terms of so-called “social capital.” The positive effect of social capital on health has been well described in previous studies [42,43]. Putnam [44] defined social capital as trust, norms, and networks that facilitate action and cooperation for mutual benefit. In this study, we did not identify a significant association between social capital in the local community and individual sleep quality. In the Multi-Ethnic Study of Atherosclerosis (MASE) carried out in the United States, higher levels of neighborhood social cohesion were associated with longer sleep duration [26]. Although this was not confirmed by a multilevel analysis, a survey by De Santis et al. [45] of 1406 individuals aged 45–84 years in six United States cities found that a lower level of social cohesion was significantly associated with shorter sleep duration. However, a study of local residents in South Korea and Taiwan by Nomura et al. [18] did not identify any associations between social capital and sleep. Few studies have addressed the association between these two factors, and only a limited number have applied multilevel analyses in particular. Further studies on such associations are required in the future.

It is known that health disparity is influenced by environmental differences. For instance, there is a two-year shorter life expectancy in the Adachi ward of Tokyo compared to the average life span in the entire city of Tokyo, based on 2010 data. An intervention to change diet habits in the Adachi ward by increasing accessibility to fresh vegetables, reduced this life span gap due to a decrease in the prevalence of diabetes. Similarly, environmental changes that increase opportunities for walking and provide multiple destinations might improve the quality of sleep in older adults.

Especially for older people who spend most of their time in residential neighborhoods, improving the environment is an important measure. In Japan, a movement is underway to open salons in local neighborhoods to encourage older people to go out [21,46]. Interventions with the objective of improving access to restaurants, small shops, and other non-residential facilities in the community are also believed to help maintain older people’s physical activity levels and prevent their health from deteriorating by encouraging them to leave the house on a regular basis [47,48]. Our results suggest that increasing the number of places in residential neighborhoods where older people feel free to drop in and improving the physical environment might improve their sleep quality.

Individual-level factors associated with sleep quality in non-depressive respondents included female sex, living alone, poor self-rated health, low income, unemployment, short walking time, and undergoing medical treatment for disease, all of which increased the risk of poor sleep quality. These results were consistent with those of previous studies, which have identified associations between sleep quality and individual-level factors including sex, income, and household composition [45]. In this study, non-depressive respondents with lower incomes slept more poorly, a finding consistent with those of previous studies [49,50]. The association between income and health is well known. Individuals with lower incomes have been found to be at higher risk of lifestyle-related diseases and depression [27,51,52,53,54], and the same may be true for sleep quality. Respondents who were unemployed also had significantly poorer quality sleep. After retirement, some people may find that withdrawal from the front line of society leaves them with feelings of exclusion and isolation, leaving them unable to find meaning in life. It is possible that an unfocused, inactive lifestyle may have an adverse effect on sleep quality.

In contrast, the only two factors significantly associated with sleep quality in depressive respondents were young age and poor self-rated health. Although there were significant associations between sleep quality and a large number of individual-level factors including sex, living alone, income, employment, walking time, and medical treatment for disease in non-depressive respondents, very few such significant associations were present in depressive respondents. Although no similar results have previously been published, as depression and sleep are closely related [55,56], the effect of the other factors may have been relatively smaller and thus evident in the analysis results.

An association between living alone and poor sleep quality has also been demonstrated in a previous study of older people living in a Chinese city [11]. The number of single-person households is projected to further increase in future. Sleep support for older people living alone will become an important issue. In this study, we found that a higher proportion of older respondents reported good-quality sleep. Previous studies have found that sleep quality deteriorates with age [11]. One possible reason for this discrepancy may be that our study participants were all aged ≥65 years, and our results therefore reflect a comparison between older people. Our study also did not include any older people registered as requiring long-term care. The particular elderly age groups in our study may therefore be comprised of older people who have maintained their health into old age. The fact that sleep quality improved with age in our study may thus have been due to the effect of “survival bias.” We did not identify any association between education attainments and sleep quality. A study of self-care activities by older people in the United States [57], as well as another of older people living in a rural community in South Korea [58], found that the lower the level of education attainment, the higher the rate of insomnia. However, an Iranian study of sleep quality in healthy older people found no association between the prevalence of sleep disorder and the highest level of educational attainment [59]. Thus, the association between education attainment and sleep quality is inconsistent. Japanese people now aged ≥80 years belong to the generation that experienced the war during their school years, and their life courses encompass experiences that differ from those of other generations [60]. This may be the reason for the absence of any association between the highest level of education attainment and sleep quality in our participants. Further, it is not a general rule that people with higher education attainment sleep better.

Reduced sleep quality in older people not only leads to lifestyle-related disorders such as hypertension and diabetes as well as depression and other mental disorders, but also reduces the quality of life (QOL) and is a contributing factor to the need for long-term care [61,62,63]. Measures to improve sleep are also needed in order to maintain the QOL of older individuals.

### Strengths and Limitations of the Study

The JAGES study data used in our analysis have provided numerous findings that might form the basis for strategies to help prevent older people from needing long-term care. Examples include the associations between social capital and neighborhood walkability [34], childhood socioeconomic status and fruit and vegetable intake, [64] and eating alone and depression [24]. This study yielded novel findings from the perspective of helping people to sleep better at the neighborhood level.

However, this study has several limitations. First, since this was a cross sectional study, we were not able to infer causality. Second, the assessment of sleep quality in this study was subjective; to further improve the reliability of our findings, it may be necessary to objectively evaluate the quality of sleep. Third, we did not consider comorbidities, which may be a confounder. The main limitation in our study was the reliance on self-reports of sleep difficulties. However, the self-reported sleep difficulties may in any case be relevant to their well-being. Fourth, the present analysis was based on data from 2010; since then, the circumstances in parts of Japan have changed drastically, particularly after the East Japan Great Earthquake in 2011. Despite this limitation, the findings obtained from the cross-sectional analysis in 2010 are still relevant to the development of current public health policy.

## 5. Conclusions

Sleep problems are closely associated with individual-level factors such as individual lifestyle habits, income, and employment. Our results suggested that in addition to these individual factors, approaching environmental factors from further upstream at the neighborhood level from the perspective of social determinants of health may also help to improve the sleep quality of local residents.

## Figures and Tables

**Table 1:** Characteristics of the study participants divided by sleep quality (good or poor).

Characteristic	Variable	Good (n = 12,579)		Poor (n = 4071)		
		n	%	n	%	*p* *
Sex	male	6227	49.5	1875	46.06	<0.001
	female	6352	50.5	2196	53.94	
Age (years)	65–69	3948	31.39	1356	33.31	0.002
	70–74	3662	29.11	1249	30.68	
	75–79	2663	21.17	807	19.82	
	80–84	1556	12.37	446	10.96	
	≥85	750	5.96	213	5.23	
Living alone	no	11,089	88.15	3460	84.99	<0.001 †
	yes	1355	10.77	562	13.8	
	missing	135	1.07	49	1.2	
Self-rated health	fair	10,729	85.29	2732	67.11	<0.001 †
	poor	1668	13.26	1276	31.34	
	missing	182	1.45	63	1.55	
Job	having	2996	23.82	757	18.59	<0.001 †
	no	8581	68.22	2969	72.93	
	missing	1002	7.97	345	8.47	
Equivalent income	<200	6783	53.92	2525	62.02	<0.001 †
(million yen)	200–400	4402	34.99	1244	30.56	
	≥400	1394	11.08	302	7.42	
Education (year)	<6	247	1.96	105	2.58	<0.001
	6–9	5375	42.73	1868	45.89	
	10–12	4517	35.91	1377	33.82	
	≥13	2256	17.93	641	15.75	
	other	59	0.47	32	0.79	
	missing	125	0.99	48	1.18	
Walking time (min)	<60	7780	61.85	2778	68.24	<0.001 †
	≥60	4162	33.09	1082	26.58	
	missing	637	5.06	211	5.18	
Treatment	yes	8378	66.6	2917	71.65	<0.001 †
	no	3316	26.36	843	20.71	
	missing	885	7.04	311	7.64	
Depressive status	GDS score of <5	10,183	80.95	2286	56.15	<0.001
	GDS score of ≥5	2396	19.05	1785	43.85	
Volunteer group	≥ 1/month	8818	70.1	2931	72	0.027 †
	< 1/month	1198	9.52	338	8.3	
	missing	2563	20.38	802	19.7	
Sports group	≥ 1/month	7930	63.04	2720	66.81	<0.001 †
	< 1/month	2540	20.19	660	16.21	
	missing	2109	16.77	691	16.97	
Hobby activity	≥ 1/month	6675	53.06	2305	56.62	<0.001 †
	< 1/month	4074	32.39	1173	28.81	
	missing	1830	14.55	593	14.57	
Community trust	very	9136	72.63	2528	62.1	<0.001 †
	slightly	2931	23.3	1400	34.39	
	missing	512	4.07	143	3.51	
Norms of reciprocity	very	7374	58.62	1959	48.12	<0.001 †
	slightly	4652	36.98	1967	48.32	
	missing	553	4.4	145	3.56	
Community attachment	very	10,590	84.19	3023	74.26	<0.001 †
	slightly	1783	14.17	1003	24.64	
	missing	206	1.64	45	1.11	
Receive emotional support	no	11,338	90.13	3574	87.79	<0.001 †
	any one	611	4.86	325	7.98	
	missing	630	5.01	172	4.23	
Provide emotional support	no	11,115	88.36	3503	86.05	<0.001 †
	any one	781	6.21	365	8.97	
	missing	683	5.43	203	4.99	
Receive instrumental	no	11,607	92.27	3633	89.24	<0.001 †
support	any one	415	3.3	289	7.1	
	missing	557	4.43	149	3.66	
Locations with graffiti or garbage	present	3349	26.62	1246	30.61	<0.001 †
	absent	9009	71.62	2764	67.89	
	missing	221	1.76	61	1.5	
Parks/foot paths suitable for exercise/walking	present	9009	71.62	2629	64.58	<0.001 †
	absent	3419	27.18	1408	34.59	
	missing	151	1.2	34	0.84	
Locations difficult for walking (hills or steps)	present	4939	39.26	1722	42.3	0.002 †
	absent	7523	59.81	2316	56.89	
	missing	117	0.93	33	0.81	
Roads/crossroads with risk of traffic accidents	present	8152	64.81	2792	68.58	<0.001 †
	absent	4291	34.11	1239	30.43	
	missing	136	1.08	40	0.98	
Fascinating views	present	5088	40.45	1378	33.85	<0.001 †
or buildings	absent	7298	58.02	2636	64.75	
	missing	193	1.53	57	1.4	
Shops or facilities selling	present	9427	74.94	2816	69.17	<0.001 †
fresh fruits & vegetables	absent	3006	23.9	1229	30.19	
	missing	146	1.16	26	0.64	
Dangerous places for	present	7444	59.18	2552	62.69	<0.001 †
walking alone at night	absent	4973	39.53	1473	36.18	
	missing	162	1.29	46	1.13	
Houses or facilities where you feel free to drop in	present	5279	41.97	1337	32.84	<0.001 †
	absent	7137	56.74	2682	65.88	
	missing	163	1.3	52	1.28	

* Chi-square test. † Scheffe’s multiple comparison procedure; Significant difference was found between GDS score of <5 living alone: no and yes (*p* < 0.001), Self-rated health: fair and poor (*p* < 0.001), poor and missing (*p* < 0.001),Job: having and no (*p* < 0.001), having and missing(*p* < 0.001), Equivalent income: <200 million yen and 200–400 million yen (*p* < 0.001), <200 million yen and ≥400 million yen (*p* < 0.001), 200–400 million yen and ≥400 million yen (*p* = 0.002), Walking time: <60min and ≥60 min (*p* < 0.001), ≥60min and missing (*p* = 0.028), Treatment: yes and no (*p* < 0.001), no and missing (*p* < 0.001),depressive status: GDS score of <5 and GDS score of ≥5: *p* < 0.001),Volunteer group: ≥ 1/month and <1/month (*p* = 0.042),Sports group:≥ 1/month and < 1/month (*p* < 0.001), 1/month and <missing (*p* = 0.001), Hobby activity: ≥1/month and <1/month (*p* < 0.001), Community trus: t very and slightly (*p* < 0.001), slightly and missing (*p* < 0.001), Norms of reciprocity: very and slightly (*p* < 0.001), slightly and missing (*p* < 0.001)), Community attachment: very and slightly (*p* < 0.001), slightly and missing (*p* < 0.001), Receive emotional support: no and any one (*p* < 0.001), no and missing (*p* < 0.001), Provide emotional support: no and any one (*p* < 0.001), no and missing (*p* < 0.001),Receive instrumental support: no and any one (*p* < 0.001), no and missing (*p* < 0.001), Locations with graffiti or garbage: present and absent (*p* < 0.001), and absent and missing (*p* < 0.001), Parks/foot paths suitable for exercise/walking: present and absent (*p* < 0.001), absent and missing (*p* = 0.004), Roads/crossroads with risk of traffic accidents: present and absent (*p* < 0.001), Fascinating views or buildings: present and absent (*p* < 0.001), Shops or facilities selling fresh fruits & vegetables: present and absent (*p* < 0.001), absent and no (*p* < 0.001), Dangerous places for Walking: alone at night: present and absent (*p* < 0.001), Houses or facilities where you feel free to drop in: present and absent (*p* < 0.001).

**Table 2:** Characteristics of participants divided by depressive status (GDS score of ≥5 or <5) and sleep quality (good or poor).

		GDS Score of <5 (n = 12,469)					GDS Score of ≥5 (n = 4181)				
		Good (n = 10,183)		Poor (n = 2286)			Good (n = 2396)		Poor (n = 1785)		
		n	%	n	%	*p* *	n	%	n	%	*p* *
Sex	male	5029	49.4	1011	44.2	<0.001	1198	50.0	864	48.4	0.307
	female	5154	50.6	1275	55.8		1198	50.0	921	51.6	
Age (years)	65–69	3304	32.5	830	36.3	<0.001 †	644	26.9	526	29.5	0.008
	70–74	3010	29.6	723	31.6		652	27.2	526	29.5	
	75–79	2135	21.0	421	18.4		528	22.0	386	21.6	
	80–84	1206	11.8	239	10.5		350	14.6	207	11.6	
	≥85	528	5.2	73	3.2		222	9.3	140	7.8	
Living alone	no	9052	88.9	1996	87.3	0.030 †	2037	85.0	1464	82.0	0.021
	yes	1021	10.0	270	11.8		334	13.9	292	16.4	
	missing	110	1.1	20	0.9		25	1.0	29	1.6	
Self-rated health	fair	9126	89.6	1812	79.3	<0.001 †	1603	66.9	920	51.5	<0.001 †
	poor	920	9.0	443	19.4		748	31.2	833	46.7	
	missing	137	1.4	31	1.4		45	1.9	32	1.8	
Job	having	2591	25.4	487	21.3	<0.001 †	405	16.9	270	15.1	<0.001
	no	6805	66.8	1631	71.4		1776	74.1	1338	75.0	
	missing	787	7.7	168	7.4		215	9.0	177	9.9	
Equivalent income (million yen)	<200	5170	50.8	1270	55.6	<0.001 †	1613	67.3	1255	70.3	0.087
	200–400	3761	36.9	801	35.0		641	26.8	443	24.8	
	≥400	1252	12.3	215	9.4		142	5.9	87	4.9	
Education (year)	<6	162	1.6	43	1.9	0.476	85	3.6	62	3.5	0.420
	6–9	4211	41.4	975	42.7		1164	48.6	893	50.0	
	10–12	3737	36.7	834	36.5		780	32.6	543	30.4	
	≥13	84	0.8	17	0.7		41	1.7	31	1.7	
	other	1944	19.1	404	17.7		312	13.0	237	13.3	
	missing	45	0.4	13	0.6		14	0.6	19	1.1	
Walking time (min)	<60	6104	59.9	1466	64.1	<0.001 †	1676	70.0	1312	73.5	0.042
	≥60	3585	35.2	703	30.8		577	24.1	379	21.2	
	missing	494	4.9	117	5.1		143	6.0	94	5.3	
Treatment	yes	6655	65.4	1582	69.2	<0.001 †	1723	71.9	1335	74.8	0.065
	no	2835	27.8	535	23.4		481	20.1	308	17.3	
	missing	693	6.8	169	7.4		192	8.0	142	8.0	
Volunteer group	≥1/month	7083	69.6	1592	69.6	0.692	1735	72.4	1339	75.0	0.161
	<1/month	1077	10.6	253	11.1		121	5.1	85	4.8	
	missing	2023	19.9	441	19.3		540	22.5	361	20.2	
Sports group	≥1/month	6254	61.4	1427	62.4	0.572	1676	70.0	1293	72.4	0.147
	<1/month	2269	22.3	487	21.3		271	11.3	173	9.7	
	missing	1660	16.3	372	16.3		449	18.7	319	17.9	
Hobby activity	≥1/month	5157	50.6	1139	49.8	0.439	1518	63.4	1166	65.3	0.423
	<1/month	3602	35.4	840	36.8		472	19.7	333	18.7	
	missing	1424	14.0	307	13.4		406	16.9	286	16.0	
Community trust	very	7704	75.7	1598	69.9	<0.001 †	1432	59.8	930	52.1	<0.001 †
	slightly	2079	20.4	613	26.8		852	35.6	787	44.1	
	missing	400	3.9	75	3.3		112	4.7	68	3.8	
Norms of reciprocity	very	6302	61.9	1255	54.9	<0.001 †	1072	44.7	704	39.4	<0.001 †
	slightly	3451	33.9	951	41.6		1201	50.1	1016	56.9	
	missing	430	4.2	80	3.5		123	5.1	65	3.6	
Community attachment	very	8836	86.8	1884	82.4	<0.001 †	1754	73.2	1139	63.8	<0.001
	slightly	1174	11.5	382	16.7		609	25.4	621	34.8	
	missing	173	1.7	20	0.9		33	1.4	25	1.4	
Receive emotional support	no	389	3.8	92	4	0.135	222	9.3	233	13.1	<0.001 †
	any one	9285	91.2	2102	92		2053	85.7	1472	82.5	
	missing	509	5	92	4		121	5.1	80	4.5	
Provide emotional support	no	467	4.6	112	4.9	0.288	314	13.1	253	14.2	0.541
	any one	9176	90.1	2070	90.6		1939	80.9	1433	80.3	
	missing	540	5.3	104	4.6		143	6	99	5.6	
Receive instrumental support	no	244	2.4	78	3.41	0.007	171	7.1	211	11.8	<0.001 †
	any one	9500	93.29	2126	93		2107	87.9	1507	84.4	
	missing	439	4.3	82	3.6		118	4.9	67	3.8	
Locations with graffiti or garbage	present	7844	77.0	1688	73.8	0.002 †	1583	66.1	1128	63.2	0.429
	absent	2221	21.8	583	25.5		785	32.8	646	36.2	
	missing	118	1.2	15	0.7		28	1.2	11	0.6	
Parks/foot paths suitable for exercise/walking	present	7502	73.7	1589	69.5	<0.001 †	1507	62.9	1040	58.3	0.004 †
	absent	2564	25.2	683	29.9		855	35.7	725	40.6	
	missing	117	1.2	14	0.6		34	1.4	20	1.1	
Locations difficult for walking (hills or steps)	present	3859	37.9	924	40.4	0.047	1080	45.1	798	44.7	0.972
	absent	6231	61.2	1347	58.9		1292	53.9	969	54.3	
	missing	93	0.9	15	0.7		24	1.0	18	1.0	
Roads/crossroads with risk of traffic accidents	present	6518	64.0	1545	67.6	0.004 †	1634	68.2	1247	69.9	0.454
	absent	3556	34.9	723	31.6		735	30.7	516	28.9	
	missing	109	1.1	18	0.8		27	1.1	22	1.2	
Fascinating views or buildings	present	4329	42.5	874	38.2	<0.001 †	1583	66.1	1128	63.2	0.051
	absent	5701	56.0	1383	60.5		785	32.8	646	36.2	
	missing	153	1.5	29	1.3		28	1.2	11	0.6	
Shops or facilities selling fresh fruits & vegetables	present	7844	77.0	1688	73.8	<0.001 †	704	29.4	460	25.8	0.017
	absent	2221	21.8	583	25.5		1658	69.2	1303	73.0	
	missing	118	1.2	15	0.7		34	1.4	22	1.2	
Dangerous places for walking alone at night	present	7844	77.0	1688	73.8	0.017 †	1583	66.1	1128	63.2	0.643
	absent	2221	21.8	583	25.5		785	32.8	646	36.2	
	missing	118	1.2	15	0.7		28	1.2	11	0.6	
Houses or facilities where you feel free to drop in	present	4575	44.9	877	38.4	<0.001 †	704	29.4	460	25.8	0.028 †
	absent	5479	53.8	1379	60.3		1658	69.2	1303	73.0	
	missing	129	1.3	30	1.3		34	1.4	22	1.2	

* Chi-square test. † Scheffe’s multiple comparison procedure; Significant difference was found between GDS score of <5 age:65–69 and 70–74 (*p* = 0.008), 65–69 and≥ 85 (*p* < 0.001), 70–74 and ≥85 (*p* = 0.001), living alone: no and yes (*p* = 0.044), Self-rated health: fair and poor (*p* < 0.001), poor and missing (*p* < 0.001),Job: having and no (*p* < 0.001), Equivalent income: <200 million yen and 200–400 million yen (*p* = 0.015), <200 million yen and ≥400 million yen (*p* < 0.001), 200–400 million yen and ≥400 million yen (*p* = 0.044), Walking time: <60min and ≥60min (*p* < 0.001), Treatment: yes and no (*p* < 0.001),no and missing (*p* = 0.041), Community trus: t very and slightly (*p* < 0.001), slightly and missing (*p* = 0.001), Norms of reciprocity: very and slightly (*p* < 0.001), slightly and missing (*p* = 0.005), Community attachment: very and slightly (*p* < 0.001),very and missing (*p* = 0.037), slightly and missing (*p* < 0.001), Receive instrumental support: no and any one (*p* = 0.025),any one and missing (*p* = 0.008), Locations with graffiti or garbage: present and absent (*p* = 0.003), Parks/foot paths suitable for exercise/walking: present and absent (*p* < 0.001), absent and missing (*p* = 0.011), Roads/crossroads with risk of traffic accidents: present and absent (*p* = 0.008), Fascinating views or buildings: present and absent (*p* = 0.001), Shops or facilities selling fresh fruits & vegetables: present and absent (*p* = 0.001), absent and missing (*p* = 0.021), Dangerous places for Walking: alone at night: present and absent (*p* = 0.019), Houses or facilities where you feel free to drop in: present and absent (*p* < 0.001) GDS score of ≥5 Self-rated health: fair and poor (*p* < 0.001), Community trust: very and slightly (*p* < 0.001), slightly and missing (*p* = 0.030), Norms of reciprocity: very and slightly (*p* < 0.001), slightly and missing (*p* = 0.011), Receive emotional support: very and slightly (*p* = 0.001), slightly and missing (*p* = 0.024), Receive instrumental suppor: t no and any one (*p* = 0.001), Parks/foot paths suitable for exercise/walking: present and absent (*p* = 0.001), Houses or facilities where you feel free to drop in: present and absent (*p* = 0.032).

**Table 3:** Results of the multi-level Poisson regression analysis to study the association between neighborhood environment and sleep quality whole participants.

		Model 1			Model 2			Model 3			Model 4		
					PR	95% CI		PR	95% CI		PR	95% CI	
**Individual factors**													
Depressive status	GDS score of <5				ref								
	GDS score of ≥5				1.93	1.80	2.07	1.93	1.80	2.07	1.92	1.79	2.06
Age (years)	65–69				ref								
	70–74				0.96	0.89	1.04	0.96	0.89	1.04	0.96	0.89	1.04
	75–79				0.82	0.75	0.90	0.82	0.75	0.90	0.82	0.75	0.90
	80–84				0.74	0.66	0.83	0.74	0.66	0.84	0.75	0.66	0.84
	≥85				0.68	0.58	0.80	0.68	0.58	0.80	0.69	0.59	0.80
Sex	male				ref								
	female				1.12	1.05	1.19	1.12	1.05	1.19	1.12	1.05	1.19
Living alone	no				ref								
	yes				1.15	1.05	1.26	1.15	1.04	1.26	1.14	1.04	1.26
Self-rated health	fair				ref								
	poor				1.67	1.55	1.80	1.67	1.55	1.81	1.67	1.55	1.81
Equivalent income (million yen)	<200				ref								
	200–400				0.95	0.88	1.02	0.94	0.88	1.01	0.94	0.88	1.01
	≥400				0.83	0.74	0.94	0.83	0.73	0.94	0.83	0.73	0.94
Job	yes				ref								
	no				1.11	1.02	1.21	1.11	1.02	1.21	1.11	1.02	1.20
Education (years)	<6				ref								
	6–9				0.94	0.76	1.18	0.94	0.75	1.17	0.94	0.75	1.17
	10–12				0.94	0.75	1.17	0.93	0.74	1.16	0.93	0.74	1.16
	≥13				0.94	0.75	1.19	0.93	0.74	1.18	0.93	0.74	1.18
	other				1.25	0.81	1.92	1.24	0.81	1.90	1.24	0.81	1.90
Walking time (min)	<60				ref								
	≥60				0.91	0.85	0.98	0.91	0.85	0.98	0.91	0.85	0.98
Treatment	yes				ref								
	no				0.87	0.80	0.94	0.87	0.80	0.94	0.87	0.80	0.94
**Social environment**													
**(Social Capital)**													
Civic participant	every 10% increase							1.14	0.91	1.44			
Social cohesion	every 10% increase							1.00	0.80	1.25			
Reciprocity	every 10% increase							0.83	0.48	1.43			
**Physical environment**													
No location with graffiti or garbage	every 10% increase										0.83	0.51	1.33
Parks or foot paths suitable for exercise or walking	every 10% increase										1.00	0.72	1.37
No difficult locations for walking such as hills or steps	every 10% increase										0.84	0.68	1.04
No risky roads or crossroads with risk of traffic accidents	every 10% increase										0.83	0.51	1.35
Fascinating views or buildings	every 10% increase										0.99	0.73	1.33
Shops or facilities selling fresh fruits and vegetables	every 10% increase										1.09	0.82	1.45
No dangerous places for walking alone at night	every 10% increase										1.33	0.79	2.23
Houses or facilities where you feel free to drop in	every 10% increase										0.59	0.36	0.95
Intercept		0.27	0.27	0.28	0.03	-0.49	0.11	0.25	-0.09	0.60	0.14	0.02	0.26
**Random effects**													
Community-level variance (SE)		0.0004	(−0.0003)		0.0002	(−0.0003)		0.0001	(−0.0003)		2.97 × 10^−11^	(5.5 × 10^−11^)	
PCV					0.50			0.75			0.99		

PR = prevalence ratio; 95% CI = 95% confidence interval. PCV = proportional change in variance. Multi-level Poisson regression analysis: Model 1 is the null model; Model 2 is individual-level variables; Model 3 was adjusted for social, capital, and individual-level variables; Model 4 was adjusted for neighborhood, environment, and individual-level variables.

**Table 4:** Results of the multi-level Poisson regression analysis to study the association between neighborhood environment and sleep quality in non-depressive participants (Geriatric Depression Scale Score of <5).

		Model 1		Model 2			Model 3			Model 4		
				PR	95% CI		PR	95% CI		PR	95% CI	
**Individual factors**												
Age (years)	65–69			ref								
	70–74			0.94	0.85	1.04	0.96	0.87	1.07	0.94	0.85	1.05
	75–79			0.75	0.66	0.85	0.77	0.68	0.87	0.75	0.66	0.85
	80–84			0.71	0.6	0.83	0.74	0.63	0.87	0.71	0.6	0.84
	≥85			0.52	0.4	0.68	0.55	0.42	0.72	0.52	0.4	0.69
Sex	male			ref								
	female			1.16	1.06	1.27	1.15	1.05	1.26	1.17	1.07	1.28
Living alone	no			ref								
	yes			1.16	1.01	1.33	1.14	0.99	1.31	1.15	1	1.32
Self-rated health	fair			ref								
	poor			1.97	1.76	2.2	1.93	1.73	2.16	1.97	1.76	2.2
Equivalent income (million yen)	<200			ref								
	200–400			0.92	0.84	1.01	0.92	0.83	1.01	0.92	0.83	1.01
	≥400			0.82	0.71	0.95	0.82	0.71	0.96	0.82	0.7	0.95
Job	yes			ref								
	no			1.16	1.04	1.29	1.13	1.02	1.27	1.15	1.03	1.28
Education (years)	<6			ref								
	6–9			0.84	0.6	1.16	1	0.91	1.09	0.82	0.59	1.14
	10–12			0.84	0.6	1.17	1.06	0.57	1.97	0.82	0.59	1.14
	≥13			0.81	0.58	1.14	0.88	0.8	0.97	0.79	0.56	1.11
	other			0.96	0.49	1.87	0.83	0.75	0.93	0.96	0.49	1.88
Walking time (min)	<60			ref								
	≥60			0.88	0.8	0.96	0.88	0.8	0.97	0.88	0.8	0.96
Treatment	yes			ref								
	no			0.84	0.76	0.94	0.83	0.75	0.93	0.84	0.76	0.94
**Social environment**												
**(Social Capital)**												
Civic participant	every 10% increase						1.21	0.88	1.67			
Social cohesion	every 10% increase						1.04	0.77	1.4			
Reciprocity	every 10% increase						0.96	0.44	2.1			
**Physical environment**												
No location with graffiti or garbage	every 10% increase									0.68	0.36	1.29
Parks or foot paths suitable for exercise or walking	every 10% increase									0.99	0.64	1.54
No difficult locations for walking such as hills or steps	every 10% increase									0.75	0.56	0.99
No risky roads or crossroads with risk of traffic accidents	every 10% increase									0.92	0.47	1.79
Fascinating views or buildings	every 10% increase									0.98	0.66	1.47
Shops or facilities selling fresh fruits and vegetables	every 10% increase									1.21	0.82	1.78
No dangerous places for walking alone at night	every 10% increase									1.41	0.7	2.86
Houses or facilities where you feel free to drop in	every 10% increase									0.51	0.26	0.98
Intercept	0.2	0.19	0.21	0.12	0.07	0.19	0.08	0.01	0.66	0.14	0.66	0.34
**Random effects**												
Community-level variance (SE)		0.00074	(0.00038)	0.06	(0.00061)	0.00045	0.00055	(0.00044)		0.00025	(0.00041)	
PCV				0.176			0.257			0.662		

PR = prevalence ratio; 95% CI = 95% confidence interval. PCV = proportional change in variance. Multi-level Poisson regression analysis: Model 1 is the null model; Model 2 is individual-level variables; Model 3 was adjusted for social, capital, and individual-level variables; Model 4 was adjusted for neighborhood, environment, and individual-level variables.

**Table 5:** Results of the multilevel Poisson regression analysis to study the association between neighborhood environment and sleep quality in depressive participants (Geriatric Depression Scale score of ≥5).

		Model 1		Model 2			Model 3			Model 4		
				PR	95% CI		PR	95% CI		PR	95% CI	
**Individual-level**												
Age (years)	65–69			ref								
	70–74			0.99	0.87	1.12	1	0.88	1.13	0.99	0.87	1.12
	75–79			0.92	0.8	1.05	0.94	0.82	1.09	0.92	0.8	1.06
	80–84			0.79	0.67	0.94	0.83	0.7	0.99	0.8	0.67	0.95
	≥85			0.83	0.68	1.01	0.86	0.7	1.06	0.84	0.68	1.02
Sex	male			ref								
	female			1.06	0.96	1.17	1.06	0.96	1.16	1.06	0.96	1.17
Living alone	no			ref								
	yes			1.14	1	1.29	1.11	0.97	1.26	1.14	1	1.29
Self-rated health	fair			ref								
	poor			1.49	1.35	1.65	1.46	1.32	1.62	1.49	1.35	1.65
Equivalent income	<200			ref								
(million yen)	200–400			0.97	0.87	1.09	0.99	0.88	1.11	0.97	0.87	1.08
	≥400			0.86	0.69	1.08	0.89	0.71	1.11	0.86	0.69	1.07
Job	have			ref								
	no			1.04	0.91	1.19	1.04	0.91	1.19	1.04	0.91	1.19
Education (years)	<6			ref								
	6–9			1.07	0.79	1.44	1.11	0.82	1.5	1.07	0.79	1.44
	10–12			1.05	0.77	1.42	1.09	0.8	1.49	1.05	0.77	1.42
	≥13			1.12	0.82	1.54	1.15	0.84	1.6	1.12	0.82	1.55
	other			1.54	0.88	2.69	1.63	0.92	2.89	1.53	0.88	2.67
Walking time (min)	<60			ref								
	≥60			0.97	0.86	1.08	0.96	0.86	1.08	0.97	0.86	1.09
Treatment	yes			ref								
	no			0.92	0.81	1.06	0.92	0.8	1.05	0.92	0.81	1.06
**Social environment**												
**(** **Social Capital)**												
Civic participation	every 10% increase						0.98	0.7	1.38			
Social cohesion	every 10% increase						1.05	0.76	1.47			
Reciprocity	every 10% increase						0.83	0.38	1.83			
**Physical environment**												
No location with graffiti or garbage	every 10% increase									1	0.49	2.04
Parks or foot paths suitable	every 10% increase									1	0.62	1.61
for exercise or walking												
No difficult locations for	every 10% increase									0.99	0.73	1.35
walking such as hills or steps												
No risky roads or crossroads	every 10% increase									0.75	0.36	1.55
with risk of traffic accidents												
Fascinating views or buildings	every 10% increase									1.01	0.64	1.57
Shops or facilities selling	every 10% increase									0.96	0.63	1.45
fresh fruits and vegetables												
No dangerous places for	every 10% increase									1.17	0.54	2.53
walking alone at night												
Houses or facilities where	every 10% increase									0.72	0.35	1.47
you feel free to drop in												
Intercept		0.47	0.45	0.25	0.15	0.43	0.28	0.03	2.41	0.31	0.14	0.67
**Random effects**												
Community-level variance (SE)		4.6 × 10^−21^	(1.09 × 10^−20^)	9.22 × 10^−18^	(1.79 × 10^−17^)		2.04 × 10^−19^	(4.87 × 10^−19^)		(1.6 × 10^−11^)	(3.9 × 10^−19^)	
PCV				-			-			-		

PR = prevalence ratio; 95% CI = 95% confidence interval. PCV = proportional change in variance. Multilevel Poisson regression analysis: Model 1 is the null model; Model 2 is individual-level variables; Model 3 was adjusted for social, capital, and individual-level variables; Model 4 was adjusted for neighborhood environment- and individual-level variables.

## References

[1] Luyster F.F.S. (2012). Sleep: A Health Imperative. Sleep.

[2] Shin H.Y., Kang G., Kim S.W., Kim J.M., Yoon J.S., Shin I.S. (2016). Associations between sleep duration and abnormal serum lipid levels: Data from the Korean National Health and Nutrition Examination Survey (KNHANES). Sleep Med..

[3] Lin S.C. (2016). The Link of Self-Reported Insomnia Symptoms and Sleep Duration with Metabolic Syndrome: A Chinese Population-Based Study. Sleep.

[4] Baglioni C., Battagliese G., Feige B., Spiegelhalder K., Nissen C., Voderholzer U., Lombardo C., Riemann D. (2011). Insomnia as a predictor of depression: A meta-analytic evaluation of longitudinal epidemiological studies. J. Affect. Disord..

[5] Hidaka B.H. (2012). Depression as a disease of modernity: Explanations for increasing prevalence. J. Affect. Disord..

[6] Cable N., Chandola T., Aida J., Sekine M., Netuveli G. (2017). Can sleep disturbance influence changes in mental health status? Longitudinal research evidence from ageing studies in England and Japan. Sleep Med..

[7] Tafaro L., Cicconetti P., Baratta A., Brukner N., Ettorre E., Marigliano V., Cacciafesta M. (2007). Sleep quality of centenarians: Cognitive and survival implications. Arch. Gerontol. Geriatr..

[8] Suh S.W., Han J.W., Lee J.R., Byun S., Kwon S.J., Oh S.H., Lee K.H., Han G., Hong J.W., Kwak K.P. (2018). Sleep and Cognitive Decline: A Prospective Non-demented Elderly Cohort Study. Ann. Neurol..

[9] Sexton C.E., Zsoldos E., Filippini N., Griffanti L., Winkler A., Mahmood A., Allan C.L., Topiwala A., Kyle S.D., Spiegelhalder K. (2017). Associations between self-reported sleep quality and white matter in community-dwelling older adults: A prospective cohort study. Hum. Brain Mapp..

[10] Liu X., Uchiyama M., Kim K., Okawa M., Shibui K., Kudo Y., Doi Y., Minowa M., Ogihara R. (2000). Sleep loss and daytime sleepiness in the general adult population of Japan. Psychiatry Res..

[11] Luo J., Zhu G., Zhao Q., Guo Q., Meng H., Hong Z., Ding D. (2013). Prevalence and risk factors of poor sleep quality among chinese elderly in an urban community: Results from the Shanghai aging study. PLoS ONE.

[12] Cochen V., Arbus C., Soto M.E., Villars H., Tiberge M., Montemayor T., Hein C., Veccherini M.F., Onen S.H., Ghorayeb I. (2009). Sleep disorders and their impacts on healthy, dependent, and frail older adults. J. Nutr. Health Aging.

[13] Stone K.L., Xiao Q. (2018). Impact of Poor Sleep on Physical and Mental Health in Older Women. Sleep Med. Clin..

[14] WHO (2010). Adelaide Statement on Health in All Policies Moving Towards a Shared Governance for Health and Well-Being.

[15] Pugh C.R., Nguyen K.T., Gonyea J.L., Fleshner M., Watkins L.R., Maier S.F., Rudy J.W. (1999). Role of interleukin-1 beta in impairment of contextual fear conditioning caused by social isolation. Behav. Brain Res..

[16] Paine S.J., Harris R., Cormack D., Stanley J. (2016). Racial Discrimination and Ethnic Disparities in Sleep Disturbance: The 2002/03 New Zealand Health Survey. Sleep.

[17] Gu D., Sautter J., Pipkin R., Zeng Y. (2010). Sociodemographic and health correlates of sleep quality and duration among very old Chinese. Sleep.

[18] Nomura K., Yamaoka K., Nakao M., Yano E. (2010). Social determinants of self-reported sleep problems in South Korea and Taiwan. J. Psychosom. Res..

[19] Johnson D.A., Lisabeth L., Hickson D., Johnson-Lawrence V., Samdarshi T., Taylor H., Diez Roux A.V. (2016). The Social Patterning of Sleep in African Americans: Associations of Socioeconomic Position and Neighborhood Characteristics with Sleep in the Jackson Heart Study. Sleep.

[20] Bassett E., Moore S. (2014). Neighbourhood disadvantage, network capital and restless sleep: Is the association moderated by gender in urban-dwelling adults?. Soc. Sci. Med..

[21] Kondo K. (2016). Progress in Aging Epidemiology in Japan: The JAGES Project. J. Epidemiol..

[22] Kondo N. (2012). Socioeconomic disparities and health: Impacts and pathways. J. Epidemiol..

[23] Wallace M.L., Stone K., Smagula S.F., Hall M.H., Simsek B., Kado D.M., Redline S., Vo T.N., Buysse D.J., Osteoporotic Fractures in Men Study Research Group (2018). Which Sleep Health Characteristics Predict All-Cause Mortality in Older Men? An Application of Flexible Multivariable Approaches. Sleep.

[24] Tani Y., Sasaki Y., Haseda M., Kondo K., Kondo N. (2015). Eating alone and depression in older men and women by cohabitation status: The JAGES longitudinal survey. Age Ageing.

[25] Sasaki Y., Aida J., Tsuji T., Miyaguni Y., Tani Y., Koyama S., Matsuyama Y., Sato Y., Tsuboya T., Nagamine Y. (2017). Does the Type of Residential Housing Matter for Depressive Symptoms in the Aftermath of a Disaster? Insights from the Great East Japan Earthquake and Tsunami. Am. J. Epidemiol..

[26] Johnson D.A., Simonelli G., Moore K., Billings M., Mujahid M.S., Rueschman M., Kawachi I., Redline S., Diez Roux A.V., Patel S.R. (2017). The Neighborhood Social Environment and Objective Measures of Sleep in the Multi-Ethnic Study of Atherosclerosis. Sleep.

[27] Saito M., Kondo K., Kondo N., Abe A., Ojima T., Suzuki K. (2014). Relative deprivation, poverty, and subjective health: JAGES cross-sectional study. PLoS ONE.

[28] Fujihara S., Tsuji T., Miyaguni Y., Aida J., Saito M., Koyama S., Kondo K. (2019). Does Community-Level Social Capital Predict Decline in Instrumental Activities of Daily Living? A JAGES Prospective Cohort Study. Int. J. Environ. Res. Public Health.

[29] Hanibuchi T., Murata Y., Ichida Y., Hirai H., Kondo K. (2008). An evaluation of an area’s social capital by public health nurses. Jpn. J. Public Health.

[30] Zhang J., Kai F.Y. (1998). What’s the relative risk?: A method of correcting the odds ratio in cohort studies of common outcomes. JAMA.

[31] Peltzer K., Pengpid S. (2019). Loneliness correlates and associations with health variables in the general population in Indonesia. Int. J. Ment. Health Syst..

[32] Balfour J.L., Kaplan G.A. (2002). Neighborhood environment and loss of physical function in older adults: Evidence from the Alameda County Study. Am. J. Epidemiol..

[33] Murayama H., Wakui T., Arami R., Sugawara I., Yoshie S. (2012). Contextual effect of different components of social capital on health in a suburban city of the greater Tokyo area: A multilevel analysis. Soc. Sci. Med..

[34] Hanibuchi T., Kondo K., Nakaya T., Shirai K., Hirai H., Kawachi I. (2012). Does walkable mean sociable? Neighborhood determinants of social capital among older adults in Japan. Health Place.

[35] Okabe D., Tsuji T., Hanazato M., Miyaguni Y., Asada N., Kondo K. (2019). Neighborhood Walkability in Relation to Knee and Low Back Pain in Older People: A Multilevel Cross-Sectional Study from the JAGES. Int. J. Environ. Res. Public Health.

[36] Murayama H.H. (2012). Contextual effect of neighborhood environment on homebound elderly in a Japanese community. Arch. Gerontol. Geriatr..

[37] Reid K.J., Baron K.G., Lu B., Naylor E., Wolfe L., Zee P.C. (2010). Aerobic exercise improves self-reported sleep and quality of life in older adults with insomnia. Sleep Med..

[38] Driver H.S., Taylor S.R. (2000). Exercise and sleep. Sleep Med. Rev..

[39] Badger T.A. (1998). Depression, Physical Health Impairment and Service Use Among Older Adults. Public Health Nurs..

[40] Cabinet O. (2019). The Section 1st, Annual Report on the Aging Society, 2018.

[41] Ministry of Health, Labour and Welfare (2002). National Health and Nutrition Survey.

[42] Murayama H., Fujiwara Y., Kawachi I. (2012). Social capital and health: A review of prospective multilevel studies. J. Epidemiol..

[43] Kawachi I., Kennedy B.P., Glass R. (1999). Social capital and self-rated health: A contextual analysis. Am. J. Public Health.

[44] Putnam R.D. (2000). Bowling Alone: The Collapse and Revival of American Community.

[45] Desantis A.S., Diez Roux A.V., Moore K., Baron K.G., Mujahid M.S., Nieto F.J. (2013). Associations of neighborhood characteristics with sleep timing and quality: The Multi-Ethnic Study Of Atherosclerosis. Sleep.

[46] Hikichi H., Kondo N., Kondo K., Aida J., Takeda T., Kawachi I. (2015). Effect of a community intervention programme promoting social interactions on functional disability prevention for older adults: Propensity score matching and instrumental variable analyses, JAGES Taketoyo study. J. Epidemiol. Community Health.

[47] Van Cauwenberg J., De Bourdeaudhuij I., De Meester F., Van Dyck D., Salmon J., Clarys P., Deforche B. (2011). Relationship between the physical environment and physical activity in older adults: A systematic review. Health Place.

[48] Hanibuchi T., Nakaya T., Yonejima M., Honjo K. (2015). Perceived and objective measures of neighborhood walkability and physical activity among adults in japan: A multilevel analysis of a nationally representative sample. Int. J. Environ. Res. Public Health.

[49] Matsumoto S., Yamaoka K., Inoue M., Muto S., Teikyo Ishinomaki Research Group, Health and Life Revival Council in the Ishinomaki district (RCI) (2014). Social ties may play a critical role in mitigating sleep difficulties in disaster-affected communities: A cross-sectional study in the Ishinomaki area, Japan. Sleep.

[50] Stamatakis K.A., Kaplan G.A., Roberts R.E. (2007). Short Sleep Duration Across Income, Education, and Race/Ethnic Groups: Population Prevalence and Growing Disparities During 34 Years of Follow-Up. Ann. Epidemiol..

[51] Scott-Lennix J.A., Lennox R.D., Hoyle R.H. (1995). Sex—Race differences in social support and depression in older low-income adults. Structural Equation Modeling: Concepts, Issues, and Applications.

[52] Inoue Y., Stickley A., Yazawa A., Shirai K., Amemiya A., Kondo N., Kondo K., Ojima T., Hanazato M., Suzuki N. (2016). Neighborhood characteristics and cardiovascular risk among older people in Japan: Findings from the JAGES project. PLoS ONE.

[53] Haseda M., Kondo N., Ashida T., Tani Y., Takagi D., Kondo K. (2017). Community social capital, built environment, and income-based inequality in depressive symptoms among older people in Japan: An ecological study from the JAGES project. J. Epidemiol..

[54] Gero K., Kondo K., Kondo N., Shirai K., Kawachi I. (2017). Associations of relative deprivation and income rank with depressive symptoms among older adults in Japan. Soc. Sci. Med..

[55] Yu J., Rawtaer I., Fam J., Jiang M.J., Feng L., Kua E.H., Mahendran R. (2016). Sleep correlates of depression and anxiety in an elderly Asian population. Psychogeriatr. Off. J. Jpn. Psychogeriatr. Soc..

[56] Sukegawa T., Itoga M., Seno H., Miura S., Inagaki T., Saito W., Uegaki J., Miyaoka T., Momose I., Kasahara K. (2003). Sleep disturbances and depression in the elderly in Japan. Psychiatry Clin. Neurosci..

[57] Spira A.P., Kaufmann C.N., Kasper J.D., Ohayon M.M., Rebok G.W., Skidmore E., Parisi J.M., Reynolds C.F. (2014). Association between insomnia symptoms and functional status in U.S. older adults. J. Gerontol. Ser. B Psychol. Sci. Soc. Sci..

[58] Kim W.J., Joo W.T., Baek J., Sohn S.Y., Namkoong K., Youm Y., Kim H.C., Park Y.R., Chu S.H., Lee E. (2017). Factors Associated with Insomnia among the Elderly in a Korean Rural Community. Psychiatry Investig..

[59] Dehghankar L., Ghorbani A., Yekefallah L., Hajkarimbaba M., Rostampour A. (2018). Association of sleep quality with socio-demographic characteristics in elderly referred to health centers in Qazvin, Iran. Sleep Hypn..

[60] Murayama H., Fujiwara T., Tani Y., Amemiya A., Matsuyama Y., Nagamine Y., Kondo K. (2018). Long-term Impact of Childhood Disadvantage on Late-Life Functional Decline Among Older Japanese: Results From the JAGES Prospective Cohort Study.

[61] Troxel W.M., Buysse D.J., Matthews K.A., Kip K.E., Strollo P.J., Hall M., Drumheller O., Reis S.E. (2010). Sleep symptoms predict the development of the metabolic syndrome. Sleep.

[62] Reichmuth K.J., Austin D., Skatrud J.B., Young T. (2005). Association of sleep apnea and type II diabetes: A population-based study. Am. J. Respir. Crit. Care Med..

[63] Kawakami N., Takatsuka N., Shimizu H. (2004). Sleep disturbance and onset of type 2 diabetes. Diabetes Care.

[64] Yanagi N., Hata A., Kondo K., Fujiwara T. (2018). Association between childhood socioeconomic status and fruit and vegetable intake among older Japanese: The JAGES 2010 study. Prev. Med..

